# Proactive Guidance for Accurate UAV Landing on a Dynamic Platform: A Visual–Inertial Approach

**DOI:** 10.3390/s22010404

**Published:** 2022-01-05

**Authors:** Ching-Wei Chang, Li-Yu Lo, Hiu Ching Cheung, Yurong Feng, An-Shik Yang, Chih-Yung Wen, Weifeng Zhou

**Affiliations:** 1 Department of Mechanical Engineering, The Hong Kong Polytechnic University, Kowloon, Hong Kong; chingwei.chang@connect.polyu.hk (C.-W.C.); hiu-ching-athena.cheung@connect.polyu.hk (H.C.C.); 2 Department of Aeronautical and Aviation Engineering, The Hong Kong Polytechnic University, Kowloon, Hong Kong; liyu.lo@connect.polyu.hk (L.-Y.L.); yurong.feng@connect.polyu.hk (Y.F.); chihyung.wen@polyu.edu.hk (C.-Y.W.); 3 Department of Energy and Refrigerating Air-Conditioning Engineering, National Taipei University of Technology, Taipei 10608, Taiwan; asyang@ntut.edu.tw; 4 School of Professional Education and Executive Development, The Hong Kong Polytechnic University, Kowloon, Hong Kong

**Keywords:** UAV, VTOL, object tracking, deep learning, sensor fusion, kalman filter, autonomous landing, optimal trajectory

## Abstract

This work aimed to develop an autonomous system for unmanned aerial vehicles (UAVs) to land on moving platforms such as an automobile or a marine vessel, providing a promising solution for a long-endurance flight operation, a large mission coverage range, and a convenient recharging ground station. Unlike most state-of-the-art UAV landing frameworks that rely on UAV onboard computers and sensors, the proposed system fully depends on the computation unit situated on the ground vehicle/marine vessel to serve as a landing guidance system. Such a novel configuration can therefore lighten the burden of the UAV, and the computation power of the ground vehicle/marine vessel can be enhanced. In particular, we exploit a sensor fusion-based algorithm for the guidance system to perform UAV localization, whilst a control method based upon trajectory optimization is integrated. Indoor and outdoor experiments are conducted, and the results show that precise autonomous landing on a 43 cm × 43 cm platform can be performed.

## 1. Introduction

In recent years, the development and application of unmanned aerial vehicles (UAVs) have been rapid. For instance, agricultural industries, construction sectors, commercial delivery corporations, and many others are now eager to adopt aerial robots to boost their working efficiency [1,2,3]. Nevertheless, the payload of a UAV is limited by the fixed capacity of the battery, and frequent landing operations are required for battery replacement or recharging. There is hence a significant need for a system for autonomous landing of UAVs on moving platforms, which would also further increase operation efficiency.

In order to address this issue, this work set out to design a system framework for a quadrotor UAV to perform landing on unmanned ground vehicles (UGV), a field of research in which several pioneering works have been published [4,5]. For instance, DHL’s AMP Electric Vehicle has been used to test the applicability of UAV pairing with delivery trucks, where aerial robots deliver parcels that are outside of the main truck delivery route. During operation, the aerial vehicles are positioned on top of the delivery truck. After loading, the UAV schedules the route to the delivery point via GPS and take off from the moving track. Meanwhile, the truck continues its rounds. After a successful delivery, the UAV flies back to the truck for its next delivery run, where it can also wirelessly recharge its battery [6]. Obviously, with such an autonomous landing system, the delivery efficiency of a single UAV can be increased. A similar technology is also under preliminary research in Amazon Prime Air [7].

The system can be further applied to marine purposes. With a robust and reliable autonomous landing system, UAVs can conduct missions on a moving unmanned surface vehicle (USV) or ship, such as auto marine condition surveying, including the detection of detailed air quality or weather conditions above the sea [8,9]. With an automatic UAV system equipped on ships, the offshore marine devices can be deployed in a much faster manner [10,11]. In addition, the UAV system can help the maritime safety department to monitor any unidentified boats or ships in the open sea in a much faster and safer manner [12,13]. Additionally, it can also be of great assistance in maritime search and rescue (SAR) missions to assist any boat or ship under threat [14,15,16,17]. The landing system can also be seen in aerial operations. In the test in [18], for example, UAVs carried by a larger flying platform could greatly reduce the time to reach the target and dramatically increase the operation range of UAVs.

In terms of the utilized sensors in this research scope, several variations can be observed. The study in [19] utilized a DJI F450 drone equipped with an infrared (IR) camera and a laser ranging sensor, in which IR beacons were installed on a moving platform. This method has the advantage of enabling landing during the night or in low-light environments. However, the low resolution of small onboard IR cameras strictly limits the sensing distance, making the detection results unreliable over middle and long ranges. Furthermore, the small angle of view of the onboard IR camera imposes a tight restriction on the heading control of the UAV. Any disturbance that affects the heading of the UAV can cause the beacon to become out of view and lead to the termination of the landing process. Moreover, in [20], the authors tried to land a UAV on top of a moving ground vehicle with an AprilTag marker on the landing pad. Two cameras were installed on the UAV to detect the marker from far and near. However, carrying two cameras onboard a UAV will significantly reduce its payload and increase power consumption. Additionally, the hardware platform in [21] utilized a DJI Matrice 100 quadrotor, which was equipped with a DJI Guidance System including a DJI Zenmuse X3 Gimbal and Camera system, an SF30 altimeter, and a DJI Manifold onboard computer. This research demonstrated its feasibility after conducting a field test of the UAV landing on a moving vehicle. Nonetheless, the approach was deemed to be nonapplicable in most scenarios, as it may overload most quadrotors with the onboard equipment. Similarly, other systems proposed by researchers [22,23] also suffered from the problem of an overweight onboard payload.

Many of the proposed works mentioned above were vision-based systems. However, such systems may suffer from accuracy issues, as conventional computer vision techniques are applied in most systems. Therefore, utilizing object detection techniques to conduct position estimation is considered an excellent alternative to increase precision. The development of object detection has received much attention in recent decades, and the research outputs in this area have been significant. Learning-based approaches, in particular, are deemed to be breakthrough contributions within the field, such as the work presented in [24,25]. Regarding vision-based robotics systems, several studies integrating learning-based approaches have been conducted. For instance, YOLOv4 was applied to develop an autonomous UAV system for electrical and mechanical devices in [26], and a support vector machine (SVM) was utilized in [27] for a quadrotor to track and follow a moving target. Additionally, the deep learning algorithm-based tracker was further adopted to conduct a surveillance mission on a UAV system [28]. However, severe motion blur could still be seen in most of the UAV’s landings, induced by the installation of cameras on the UAV. Notably, the configuration that has been proposed in most prior research may also result in a limited field of view (FoV). 

The control module of the landing process is deemed to be one of the most important modules for a complete landing system. In particular, the trajectory of the descent to the landing pad is important, which should be varied according to the configuration of the UAV. In [29], two stages of landing were designed: (1) approaching from long range, and (2) landing after hovering above the landing platform. Moreover, a stage for the UAV to land after the approach was specifically developed; however, there was no descending trajectory for the UAV to follow. Such a preliminary landing approach could then lead to an unstable landing, as external factors such as wind disturbance may have an impact. To address such a problem, the study presented in [30] specifically focused on controller development for wind disturbance rejection during the landing process. However, their proposed system was considered to be compromised in certain scenarios, as it purely relied on artificial pattern tags for the perception module. Therefore, in the current work, an optimized landing trajectory is included and addressed to ensure the robustness of the system.

**Figure 1:**
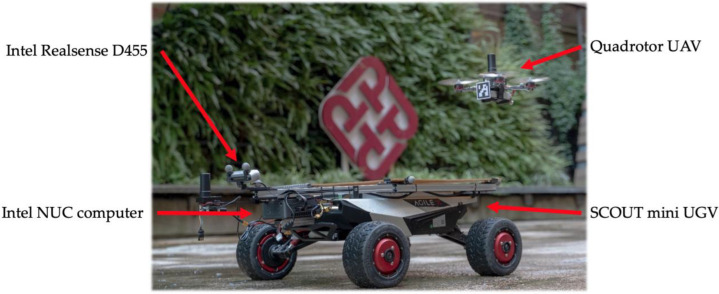
Prototype of the proactive guidance UGV and the UAV.

**Figure 2:**
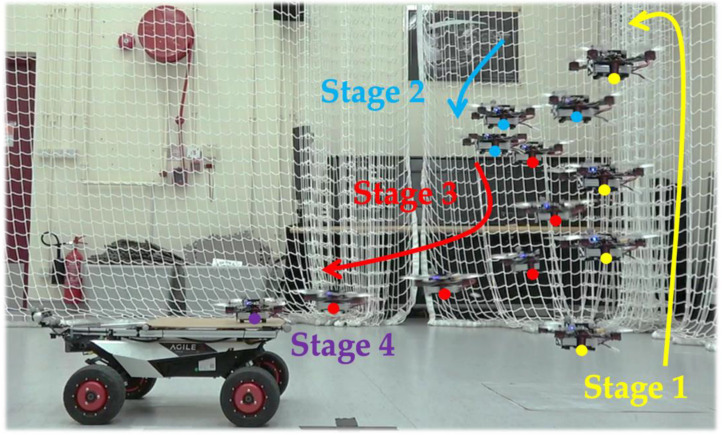
Schematic of the four stages of the automatic landing strategy.

In this study, inspired by the instrument landing system (ILS) in the real-world aviation industry, a UAV landing framework based upon a guidance system located on a UGV is proposed. We proposed a sensor-fusion and estimation method comprising multiple sources, including the ArUco marker, YOLO object detector, stereo depth information, and IMU information. A depth camera is mounted at the landing platform, as shown in Figure 1, and an ArUco tag is attached to a square pad at the front of the UAV. With this design, it is expected that the capacity of the computation unit can be increased compared to that of UAV configurations with a companion computer onboard. In the proposed system, a finite state machine is designed to track and control the landing process, as shown in Figure 2. This state machine includes four stages, which are Stage 1—GPS following, Stage 2—vision position following, Stage 3—ground-effect free trajectory, and Stage 4—shutdown. In particular, the state of the ground-effect free trajectory signifies the generation of optimized trajectories for the UAV to track and follow. It is deemed that the elucidated framework can provide a faster, smoother, and safer landing than the previous works.

The innovations of this research include the following: (1) Promoting a new installation method for the auxiliary equipment, such as the camera and the onboard computer, on a moving platform, such as the USV/UGV, instead of on the UAV itself. This arrangement can minimize the demand of the UAV payload and simultaneously maximize the usage of the computational power. (2) Proposing the application of a ground-effect free trajectory to the autolanding system, which shows a significant improvement from the ordinary vertical landing path of other research. (3) Fusing the position estimation results from IMU, YOLO, and ArUco for automatic landing systems in order to simultaneously achieve high estimation precision and a high updating rate. (4) Designing a finite state machine of the UAV to land on a moving platform for the consideration of robustness and safety.

The remainder of this paper is structured as follows: Section 2 provides the detailed system architecture, including the vision-based position detection, the UAV position estimation method, the design of the finite state machine, and the development of the trajectory planner free from the ground effect. Section 3 describes the experimental platform and test environment. Section 4 then presents the experimental results and discussion, and Section 5 concludes this work.

## 2. System Architecture

**Figure 3:**
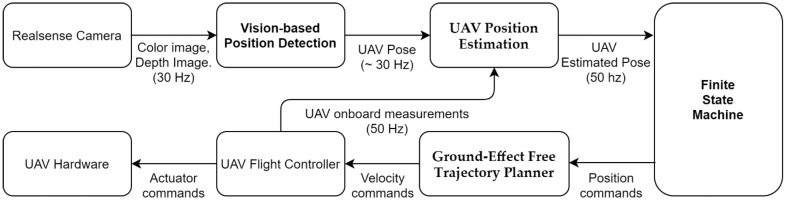
The system architecture of the autonomous landing system.

The landing system consists of several modular components, including the following: (1) vision-based position detection, (2) UAV position estimation, (3) a finite state machine, and (4) a ground-effect free trajectory planner. The relationship of each module is presented in Figure 3. This modular framework enables the use of different operating frequencies in independent modules, and can be easily modified or replaced with other algorithms.

### 2.1. Vision-Based Position Detection

In the proposed system, computer vision technologies are applied to perform vision-based landing guidance. To achieve this, the system must be able to perceive an aerial vehicle in a 3D world, which can act as a feedback signal for the control system.

Specifically, computer vision markers and a learning-based detector are used. ArUco markers are exploited and attached to the front of the quadrotor. Due to the high color contrast, the marker can be easily detected from the landing platform. By solving the Perspective-n-Point (PnP) problem, the relative position of the ArUco marker from the camera can be known. The inferred position information is then processed to acquire the position of the UAV. The major advantage of utilizing an ArUco marker is that it provides an extremely high update rate at a certain level of accuracy; however, the system suffers severely from frequent detection loss due to occasional motion blur when the UAV conducts aggressive maneuvers. When the vehicle is relatively distant or has severe illumination problems due to unstable weather, the small marker size becomes the major limitation.

**Figure 4:**
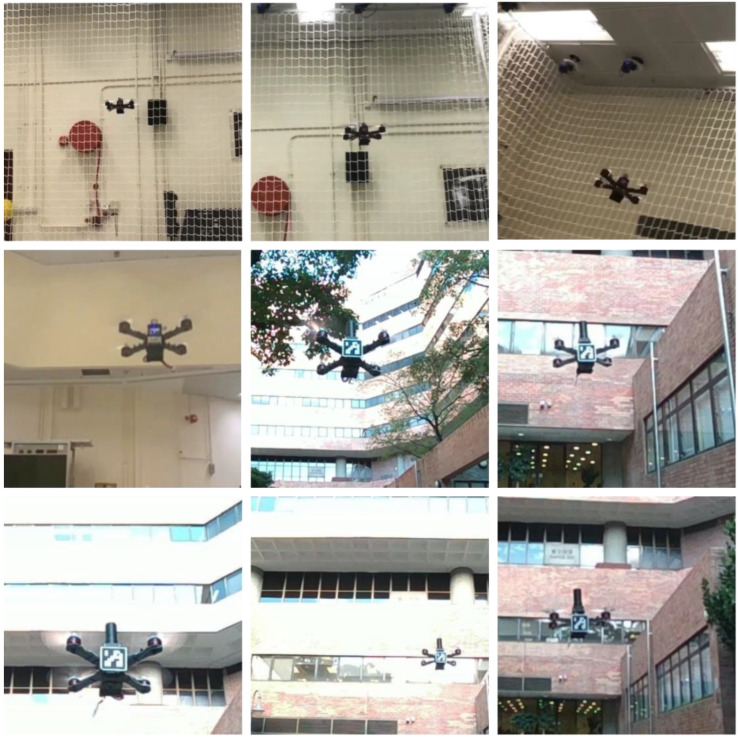
Examples of the training dataset.

Therefore, to increase the robustness, in addition to the ArUco marker, the state-of-the-art YOLOv4 algorithm is also deployed, by which the loss of detection can be addressed. To apply YOLOv4, the foremost and most crucial step is to establish a meticulously labeled dataset and conduct model training to generate a custom model. Accordingly, the dataset should consist of diverse images of the UAV under different sizes, angles, resolutions, backgrounds, and illumination conditions. Despite being relatively computationally demanding, it is deemed that the high accuracy and robustness detector can increase the stability of the proposed system. Furthermore, in real application scenarios, the moving platforms, such as automobiles or marine vessels, are capable of carrying computation facilities with powerful GPUs. It is deemed that YOLOv4 can perform UAV detection in real time, giving the system a sufficiently high update rate. Regarding the training phase of the YOLO detector, we manually labelled 2500 images, including 500 images of the validation dataset, while providing 2000 objectless background images. Specifically, the images varied in terms of the illumination conditions, angle, object size, and backgrounds. Figure 4 shows some of the training dataset for our YOLO detector.

During a landing process, both ArUco and YOLO modules first conduct predictions on the streaming frame. The ArUco detector then generates 3D position information of the observed ArUco marker, and the YOLO model produces a Region of Interest (ROI) that possibly contains the aerial vehicle. For the retrieved results from the ArUco marker, the information is in the body frame (B), and thus the 3D position of the ArUco in the world frame (W) is then calculated by the following:

(1) $$\left[\begin{array}{c} X_{i}^{W}\\ 1 \end{array}\right]=T_{B}^{W}\left[\begin{array}{c} X_{i}^{B}\\ 1 \end{array}\right],T_{B}^{W}\in SO\left(3\right),$$

where $X_{i}^{B}$ is the object position vector in the body frame (B), and $X_{i}^{W}$ is the 3D object state vector in the world frame (W). $T_{B}^{W}$ is the transformation matrix between the body frame (B) and the world frame (W).

Regarding the object detection model, the 2D bounding box is further integrated with the stereo reconstruction techniques. The depth of the pixels proximate to the center of the ROI is then obtained, followed by the calculation of the 3D position relative to the camera pose. To further acquire the 3D position information in the world frame (W), coordination transformation is then applied:

(2) $$\left[\begin{array}{c} X_{i}^{W}\\ 1 \end{array}\right]=T_{B}^{W}T_{C}^{B}\left[\begin{array}{c} X_{i}^{C}\\ 1 \end{array}\right],T_{B}^{W},\;T_{C}^{B}\in SO\left(3\right),$$

where $X_{i}^{C}$ is the object position vector retrieved in the camera frame (C).

From the limited sensors available, we further filter the two different estimations in order to retrieve the final optimal estimation. The section below discusses the details of the filter-based estimation.

### 2.2. UAV Position Estimation

As explained in the previous section, the vision-based position detection module detects and calculates the UAV’s position in the world frame (W). However, due to the limitation of the camera frame rate (max. 30 Hz, and the object is not always detectable), computational power, and sudden loss of visualization, the reliability of the position reference is not high enough for control of the UAV in some circumstances. Furthermore, having a faster estimating rate of the position also increases the response of the flight controller to provide better flying performance. To address these problems, a sensor fusion of multiple sources of position input (ArUco and YOLO Depth) and a position estimation approach were developed for the system.

To increase the updating rate and enhance the reliability of the position estimation, information from the onboard controller of the UAV is collected and implemented in the position estimating system in addition to the position information from the ArUco and YOLO depth. In this approach, a Kalman filter (KF) was designed for this position estimation task of a flying UAV. The UAV’s state vector is written as follows: $s={\left[p_{x},p_{y},p_{z},v_{x},v_{y},v_{z}\right]}^{T}$, where $p_{x},p_{y},p_{z}$ represent the position, and $v_{x},v_{y},v_{z}$ represent the velocity according to the x,y,z coordinates in the world frame (W). The UAV’s state in the system is predicted with the following model:

(3) $$s_{k}=A_{k}\;s_{k-1}+w_{k},$$

where $s_{k}$, $w_{k}$, and $A_{k}$ are the state of the system, the processing noise (which is assumed to be a white Gaussian noise), and the state transition matrix of the flying UAV, respectively. The measurement model of the system is also considered to be subject to white Gaussian noise $v_{k}$, and is expressed as:

(4) $$z_{k}=H\left(s_{k}\right)+v_{k}.$$

In the proposed system, the measurement can be either from the vision system or the onboard sensors. However, due to the different updating rates of the ArUco module, the YOLO module, and the IMU in the flight controller, data are not synchronized. To overcome this problem, the first-come-first-served algorithm is adopted. At every update stage, the filter updates the velocity measurements from the flight controller (the highest updating rate), and then checks the state from the vision system. If there are new updated position data, the filter also updates the measured position result. When the system retrieves two measured positions in the same step, it uses the result from the vision system as the measurement input.

According to the structure of the Kalman filter, the system contains two modules, namely, the prediction module and the measurement-correction module. The two modules alternate continuously. During the predicting stage, the state $s_{k}$ of the UAV is first predicted by Equation (3), in addition to the calculation of the uncertainty of the system, the covariance $C_{k},$ as:

(5) $$C_{k}=A_{k}\;C_{k-1}\;A_{k}^{T}+Q,$$

where Q is another random Gaussian noise term. In the following measurement-correcting stage, the Kalman gain $K_{k}$ is calculated as follows:

(6) $$K_{k}=C_{k}\;H^{T}\;{\left(H\;C_{k}\;H^{T}+Q\right)}^{-1}.$$

Finally, the output state $s_{k}^{-}\;$and the covariance $C_{k}^{-}$ are corrected with the Kalman gain in the following equations:

(7) $$s_{k}^{-}=s_{k}+K_{k}\left(z_{k}-H\;s_{k}\right),$$

(8) $$C_{k}^{-}=\left(I-K_{k}\;H\right)\;C_{k}.$$

### 2.3. Finite State Machine

The finite state machine (FSM) module aims to help the recovery and landing stages of an entire flying mission. At the beginning of the state machine, the state of the UAV is assumed to be under hovering mode waiting for landing. There are a total of four stages (states), including:

1. GPS following;
2. Vision position following;
3. Ground-effect free trajectory following;
4. Shutdown.

**Figure 5:**
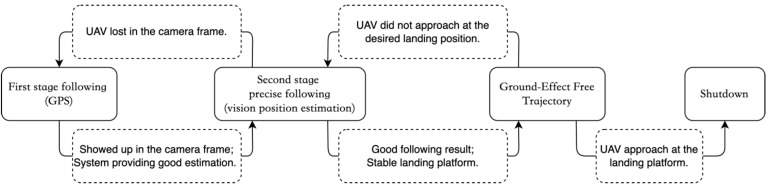
The framework of the finite state machine.

Figure 5 describes the logical connection of each state in the finite state machine and the criteria of state changing. The following subsections provide the detail.

#### 2.3.1. First Stage—GPS Following

In this first stage, the UAV is commanded to follow the landing platform at a preprogrammed distance and height difference. These parameters differ for different UAV models. The main purpose of this stage is to use the GPS, which has limited positioning accuracy, to lead the UAV to enter the FoV of the autonomous landing system. Whenever the positioning estimation module starts to provide a reasonable (or convergence) reading of the position, it triggers the state machine to switch to the next stage.

#### 2.3.2. Second Stage—Vision Position Following

After switching to this stage, the FSM begins to pass velocity but not position commands to the UAV’s flight controller. The velocity setpoints are generated by the FSM’s internal PID position (including x, y, z, and yaw) controller using the position reference from the position estimation module. The UAV continues to follow at the same desired position as the first stage, but is controlled through the proposed landing system without any GPS information. The goal of this stage is to ensure the stability of position controlling and to wait for the landing platform to be ready for approaching. When the position difference between the landing platform and the UAV is in the desired domain, the stage then proceeds to the following motion. Note that the desired domain is defined as a sphere with a radius of 0.1 m. The center of this sphere is 1.1 m behind the landing pad in the horizontal direction and 0.7 m above. Furthermore, if the UAV’s visibility is lost in the vision system and no position estimation result is provided, the system rapidly moves the stage backward to guide the UAV using GPS.

#### 2.3.3. Third Stage—Ground-Effect Free Trajectory Following

The purpose of this stage is to carry out the approaching maneuver in a smooth and safe trajectory, which generates fewer ground effects compared to landing vertically. A detailed description of the method is described in Section 2.4. When the UAV meets the landing position, the state machine then switches to the last stage. Several failsafe conditions are considered while the UAV is conducting this approaching maneuver: (1) the divergence of the UAV’s position with the desired trajectory; (2) insufficient remaining altitude of the UAV to meet the final approach’s requirement; and (3) the amount of overshoot of the UAV’s position to the landing platform. If any of these criteria are out of bounds, the state machine immediately switches back to the previous stage and rapidly separates the UAV from the landing platform to maintain a safe distance.

#### 2.3.4. Fourth Stage—Shutdown

The final stage is used to ensure the safety and stability of the UAV upon its contact with the landing platform. As the UAV enters the landing platform, ground effects start to affect the stability of the UAV. When the UAV moves toward the landing platform within a reasonable distance (<5 cm in this study) above the landing pad, the FSM commands the UAV to drastically reduce the throttles of the motors until the motors are all shut down, after which the UAV lands solidly on the platform.

### 2.4. Ground-Effect Free Trajectory Planner

In this section, the landing trajectory planner is introduced. There are two main purposes of this trajectory planner: (1) ensuring the smoothness and continuity of the approaching maneuver; and (2) minimizing the instability caused by the ground effect. 

Based on the work in [31], in which a super computational efficient optimal spatial–temporal trajectory planner was proposed, we developed our method to ensure the smoothness throughout the approaching and landing maneuver. This method can improve the computational efficiency, which enables this planner to generate a trajectory within a few microseconds. Moreover, by setting up the parameters with the trajectory generator, the physical actuation constraints of the UAV can be easily secured.

To reduce the significant ground effect and aerodynamic drag from the UAV, the desired trajectory is expected to reduce its vertical movements only. In the proposed trajectory, the UAV is expected to rapidly reduce its altitude in open air before gliding onto the landing pad to prevent the possibility of causing ground effects. Therefore, the Brachistochrone curve, which has the characteristic of using the shortest time to move an object with gravity from a higher point to a lower destination, is introduced to this trajectory planner. The equation of the curve is written as:

(9) $$\begin{array}{c} Horizontal\;path=\frac{\alpha }{2}\left(\theta -\sin\theta \right)\;,\\ Vertical\;path=-\frac{\alpha }{2}\left(1-\cos\theta \right),\\ 0<\theta <\pi . \end{array}$$

where α is the altitude difference between the UAV and landing pad. The proposed trajectory considers the initial state of the UAV and the landing pad’s dynamic to generate a series of discrete waypoints according to the Brachistochrone curve.

## 3. Experimental Architecture

To validate the proposed UAV system, both indoor and outdoor experiments were carried out. In this section, the detailed system architecture and the test environment are introduced.

### 3.1. Landing Platform Hardware Design

**Figure 6:**
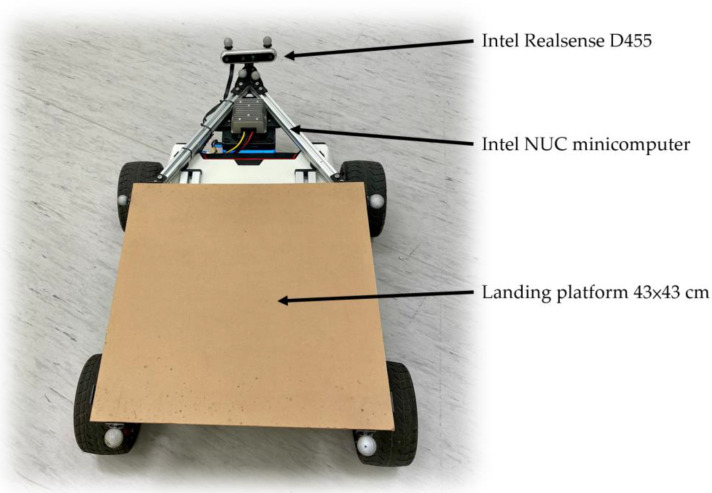
The hardware setup of the UGV landing platform.

In the experiments, a small Scout Mini (AgileX Robotics, Shenzhen, China) UGV was chosen to be the moving platform of the system. Figure 6 shows the setup of the UGV. An acrylic square pad (43 × 43 cm) was mounted directly on the top of the vehicle, and a set of Intel RealSense D455 stereo cameras (Intel Corporation, Austin, TX, USA) was fixed in front of the landing platform with an upward-facing angle of 20°. The cameras were connected to an Intel NUC minicomputer (NUC8i7BEH, Intel Corporation, USA) for image collecting and processing.

### 3.2. UAV Hardware Design

**Figure 7:**
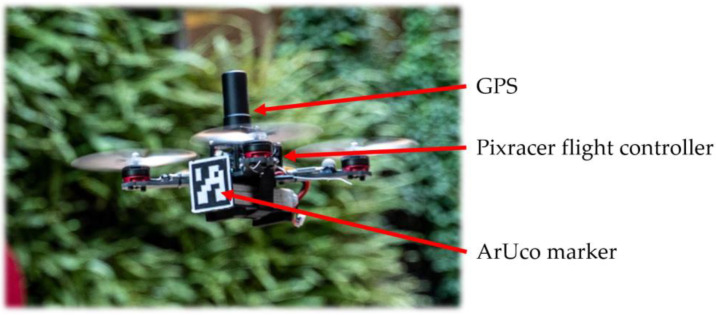
The hardware setup of the quadrotor UAV.

Figure 7 shows the UAV applied in the flight tests, which is a typical X-type quadrotor with a diameter of 210 mm. The four motors are EMAX RS2205 (EMAX model, Shenzhen, China) controlled through four T-MOTOR F35A (T-MOTOR, Nanchang, China) Electronic Speed Controllers (ESC). The ESCs are commanded by the flight controller, which is a mRo Pixracer (Mayan Robotics LLC, Chula Vista, CA, USA). The flight controller is equipped with several onboard sensors, including a gyroscope, accelerometer, compass, and barometer, to self-control its attitude during the flight. In addition, The Pixracer is also equipped with an onboard WIFI module, which can directly connect to the ROS system with MAVROS. This small quadrotor is also equipped with a front facing 3D-printed ArUco plate (45 × 45 mm) to be recognized by the vision-based system.

### 3.3. Test Environment

**Figure 8:**
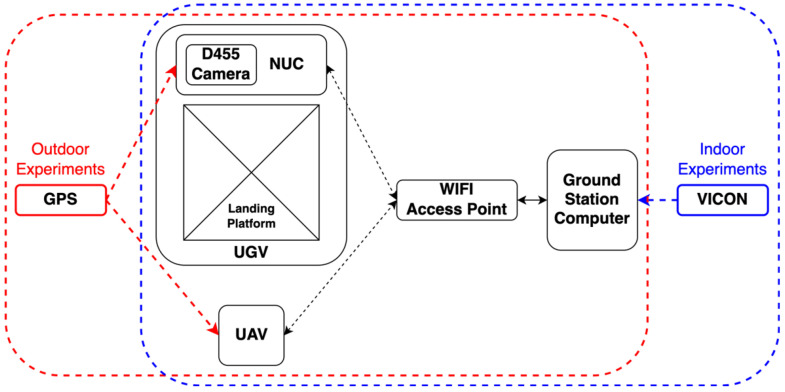
The framework of the experimental setup.

In Figure 8, the framework of the experimental system is demonstrated. The landing platform and UAV are connected through a WIFI access point to the ground station computer, which also acts as the major computational center. This ground station computer is equipped with an Intel I7-10700KF (Intel Corporation, Austin, TX, USA) processor and two NVIDIA RTX 3090 (Nvidia Corporation, Santa Clara, CA, USA) Graphics Processing Units (GPU), ensuring the computational capacity of the system. The flight experiments were first held in an indoor laboratory (blue), which was equipped with a VICON (Vicon Motion Systems, Oxford, UK) motion capture system providing the real-time ground truth information to the ground station computer. Then, the experiments were moved to the outdoors for testing the system in a real-world environment (red). During the outdoor tests, individual GPS modules were placed on both UGV and UAV, such that they could be guided roughly by the GPS coordinates information.

Notably, the current setup only served as a demonstration. The UGV, which was the current experimental platform, was unable to carry a full-size computer onboard. The ground station computer was remotely connected to the UAV and UGV through a high bandwidth wireless network. In future real applications, the ground station can be directly installed on the landing platform with multiple cameras linking to it, providing seamless and powerful computational power.

## 4. Results and Discussion

This section presents and discusses a series of indoor experiments and outdoor flight experiments.

### 4.1. Indoor Experiment Results

This section summarizes the experiment results that were conducted in a controlled indoor environment. Ground truth data from the VICON system were used to validate the proposed vision-based position estimation result. The proposed trajectory generating method and conventional vertical landing method were also compared.

#### 4.1.1. Vision-Based Position Estimation Validation

**Figure 9:**
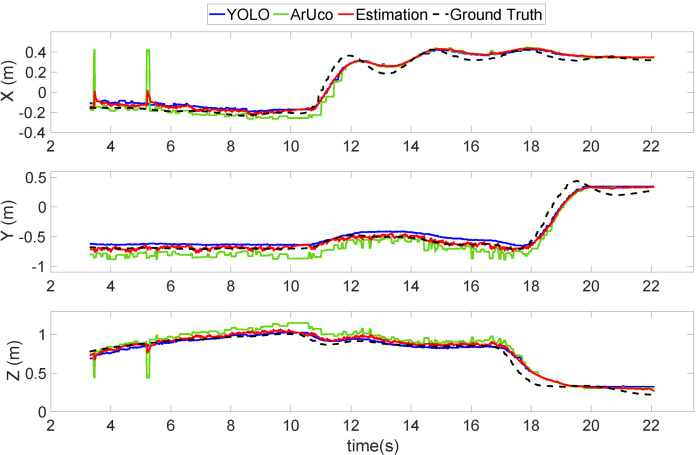
Flight test 1—Position estimation results using the proposed vision-based system and sensor fusion method in a moving platform landing mission.

In this section, two flight tests were conducted with the quadrotor UAV in the controlled laboratory for validating the position estimation performance. The first flight test, shown in Figure 9, demonstrated the landing of the quadrotor UAV on the UGV. Figure 9 shows that, at the beginning of the mission, the estimation results from the ArUco (green line) indicated two outliers. When the UAV approached the UGV, the estimation results from both the ArUco and YOLO (blue line) tightly followed the ground truth value (black dashed line). By applying sensor fusion, the estimation results (red line) could precisely follow the ground truth value during the whole experiment.

**Figure 10:**
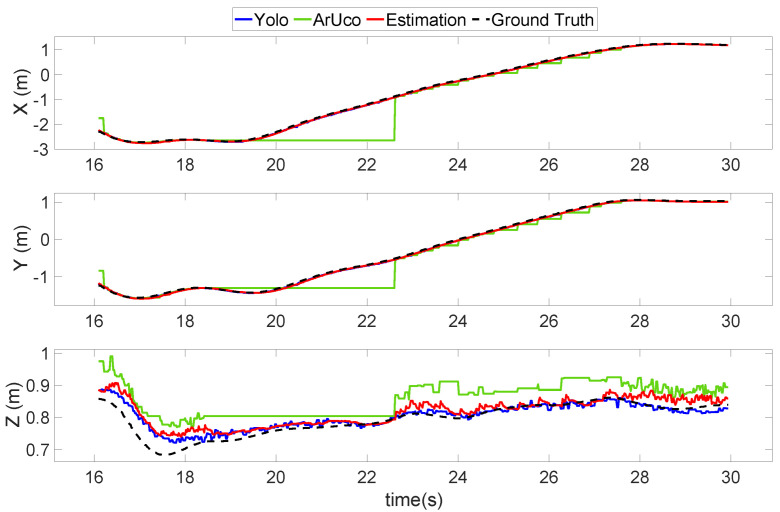
Flight test 2—Special case of position estimation using the proposed vision-based system to demonstrate the drawbacks of the ArUco and the advantages of the sensor fusion method.

Figure 10 shows the second validation flight test, which was a scenario designed for the UAV to follow the landing platform with a large separation. Some unwanted straight horizontal lines are shown in the ArUco state (green line) in Figure 10, especially from 18.5 to 22.5 s. Additionally, its performance in the z-direction was the worst among all directions throughout the entire experiment. These straight horizontal lines indicate that, in these time segments, the ArUco marker on the UAV was not recognized by the vision position detector on the UGV. This was mainly because the ArUco marker was relatively small in the captured image and, as a result, it was difficult for the ArUco algorithm to recognize the marker. Therefore, the ArUco estimation result in this circumstance was unstable and inaccurate.

By comparison, the YOLO (blue line) result maintained a very close distance to the ground truth (black dashed line), which shows that the proposed learning-based system has a much more stable and accurate recognition ability. With the help of the YOLO, the overall estimation (red line) was able to maintain a tight track to the ground truth throughout the experiment period. This result shows that it is critical to include learning-based recognition in the system for large separation situations.

However, this does not mean that the ArUco estimation method should be abandoned. In small separation situations, the ArUco estimation method plays an important role. When the UAV is very close to the landing pad, the view of the UAV in the camera may be too large to be recognized by the YOLO. Moreover, whenever there are small relative movements, the UAV can easily fall out of the field of view. The position estimated by YOLO will be unstable and inaccurate in this type of circumstance, as shown in Section 4. The proposed sensor fusion method can reject inaccurate results from the ArUco or YOLO estimations, resulting in a reliable and precise position estimation.

Table 1 displays the root mean square error (RMSE) of the estimated position to the ground truth, which shows that the overall performance of the position estimator was stable and precise enough to perform the auto landing task. The overall error was less than 8 cm compared to the size of the landing platform of 43 cm. Therefore, the proposed estimation system is precise enough to perform the autonomous landing task.

#### 4.1.2. Landing Trajectories Experiments

As presented in this section, the UAV was commanded to perform two kinds of landing trajectories toward a non-moving landing platform. These experiments were also conducted in the controlled VICON laboratory, where the position reference was directly detected and provided by the VICON system for accurate validation.

**Figure 11:**
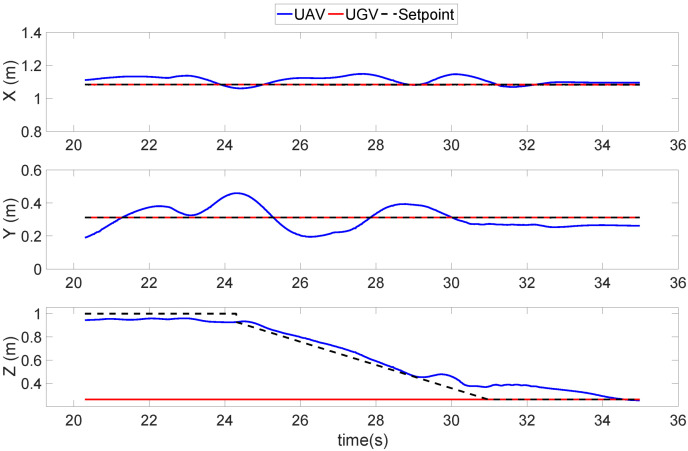
Position results of a quadrotor UAV conducting a conventional vertical landing trajectory.

Figure 11 shows the first landing trajectory experiment, which was a conventional vertical landing trajectory. As the figure shows, while the UAV started to move downward vertically, its horizontal position started to wobble, and it was unable to precisely maintain the commanded setpoint. Furthermore, during the last 15 cm (29–34 s) before touching down in the z-direction, the UAV had great difficulty in maintaining its descent because the ground effect began to take effect. In this specific period of time, the horizontal position in the x- and y-directions was even more unstable.

**Figure 12:**
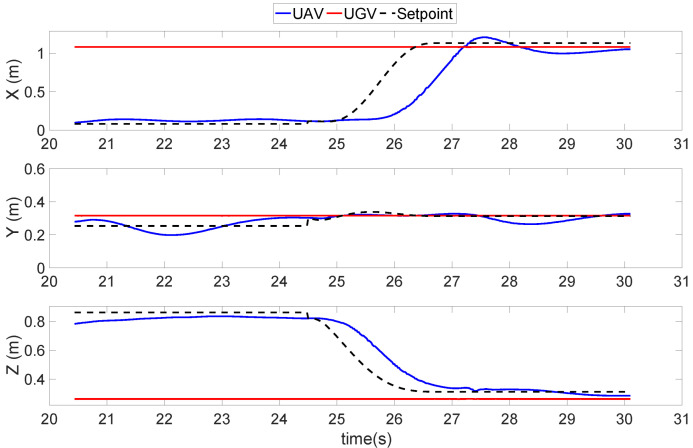
Position results of a quadrotor UAV conducting the proposed ground-effect free landing trajectory.

On the contrary, the experiment shown in Figure 12 applied the proposed ground-effect free trajectory. The UAV entered the landing state at an initial position of 1 m from the AGV in the x-direction and 0.25 m in the y-direction. Then, the UAV started to follow the gliding and landing trajectory, which descended in the z-direction while also approaching the AGV in the x- and y-directions. The effectiveness of the ground effect was shown to gradually reduce in the final period. Additionally, the horizontal position performance was also better than the previous results shown in Figure 11.

### 4.2. Outdoor Experiment Results

These experiments tested the system in a real-world environment. In this flight test, the quadrotor UAV was trying to land on the UGV, which was moving at a constant speed, and the functionality of the state machine was validated. In this outdoor experiment, the threshold of the UAV’s position to the landing platform was 5 cm above the landing pad (in the vertical direction) and 15 cm from the center line of the landing platform (on the horizontal plane). The position of the UGV was determined by the onboard GPS, and the position of the UAV was derived from the proposed position estimating system.

**Figure 13:**
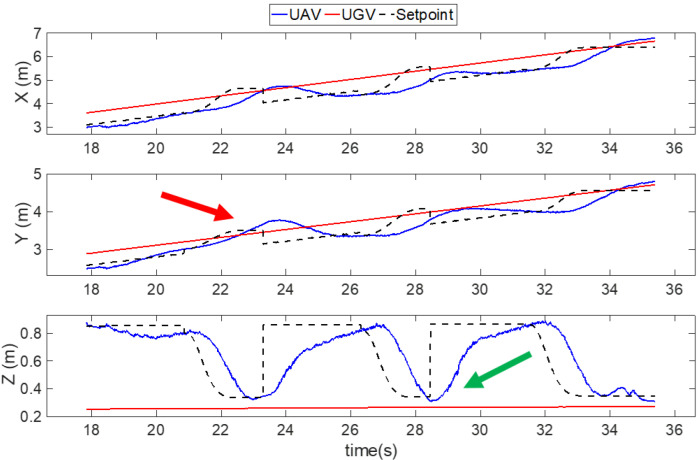
Position results of the UAV landing on a moving platform in an outdoor experiment. The red and green arrow show the overshoots of the UAV in experiment, which cause the failsafe to trigger.

Figure 13 shows that the UAV made three attempts to approach the landing pad, which were at 20.8–23.3 s, 26.3–28.4 s, and 31.5–35 s. In the first attempt, the state machine entered the third stage—ground-effect free trajectory following at 20.8 s. During the approach maneuver (20.8–23.3 s), the positions in the x- and z-directions were well followed with only one second of delay. However, there was an overshoot in the y-direction at 24 s (red arrow), which triggered the failsafe and rewound the state machine to the second stage—vision position following.

The second attempt (26.3–28.4 s) showed a similar phenomenon; however, an overshoot occurred in the z-direction at 28.5s (green arrow), which means the altitude of the UAV dropped beneath the expected level. The state machine also switched back to the second stage immediately. In the third attempt (31.5–35 s), the UAV finally met all the safety requirements and landed safely on the landing platform.

**Figure 14:**
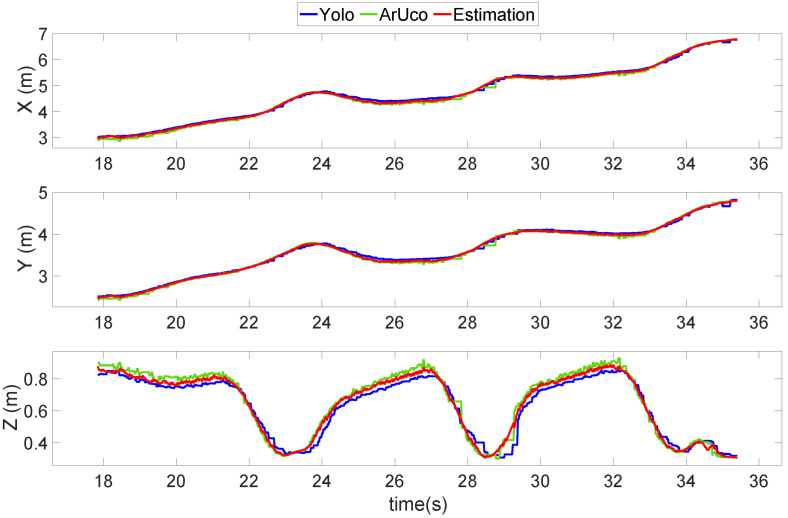
Position estimation results of the UAV landing on a moving platform in an outdoor experiment.

Furthermore, Figure 14 shows the results from the vision system. The figure shows that, particularly during the second approach (27–30 s), in which the UAV was very close to the camera, the YOLO (blue line) started to lose track of the UAV. At the same time, the ArUco was functioning appropriately, which kept the position estimation results on track. Hence, these results show the importance of sensor fusion and redundancy in the vision system.

## 5. Conclusions

In this study, the objective was to develop a proactive guidance system for UAVs to land accurately on a moving platform. As a result of the proposed system structure, the UAV’s onboard instruments can be greatly reduced. Two sets of vision-based position detection modules were first introduced, followed by sensor fusion and estimation techniques aimed at enhancing position estimation. Next, a finite state machine system with an optimal trajectory generating method was designed to accomplish this landing guidance task.

Flight experiments were undertaken in both indoor and outdoor environments. The vision-based estimation results were first validated and tested in a controlled laboratory. The results demonstrated the reliability of the vision-based system. In the landing trajectory experiments, a feasible means to prevent the unstable movement caused by ground-effects during the landing progress was developed. Finally, the system was tested in a real-world environment, in which the UAV safely landed.

To further improve the UAV landing guidance system, attention will focus on the estimation of the relative movement of the UAV and the landing platform. In marine applications, the landing platform is expected to heave and shake on waves. The system must be able to accurately measure and rapidly estimate the relative movements to achieve a safe landing in a more unstable environment.

The following are available online at https://youtu.be/OfGBQfCveiM (accessed on 1 December 2021), Video: Proactive Guidance for Accurate UAV Landing on a Dynamic Platform: A Visual–Inertial Approach. 

## Figures and Tables

**Table 1:** Calculated RMSE of UAV position estimation.

Error Evaluation	X (m)	Y (m)	Z (m)
Flight test 1	0.0437	0.0747	0.0536
Flight test 2	0.0335	0.0275	0.0312

## Data Availability

Not applicable.
